# Propensity Score-Matched Analysis of Open Surgical and Endovascular Repair for Type B Aortic Dissection

**DOI:** 10.1155/2011/364046

**Published:** 2011-07-20

**Authors:** Michael E. Brunt, Natalia N. Egorova, Alan J. Moskowitz

**Affiliations:** Department of Health Evidence and Policy, Mount Sinai School of Medicine, One Gustave L. Levy Place, P.O. Box 1077, New York, NY 10029, USA

## Abstract

*Objective*. To identify national outcomes of thoracic endovascular aortic repair (TEVAR) for type B aortic dissections (TBADs). *Methods*. The Nationwide Inpatient Sample database was examined from 2005 to 2008 using ICD-9 codes to identify patients with TBAD who underwent TEVAR or open surgical repair. We constructed separate propensity models for emergently and electively admitted patients and calculated mortality and complication rates for propensity score-matched cohorts of TEVAR and open repair patients. *Results*. In-hospital mortality was significantly higher following open repair than TEVAR (17.5% versus 10.8%, *P* = .045) in emergently admitted TBAD. There was no in-hospital mortality difference between open repair and TEVAR (5.6% versus 3.3%, *P* = .464) for elective admissions. Hospitals performing thirty or more TEVAR procedures annually had lower mortality for emergent TBAD than hospitals with fewer than thirty procedures. *Conclusions*. TEVAR produces better in-hospital outcomes in emergent TBAD than open repair, but further longitudinal analysis is required.

## 1. Background

Aortic dissection is a rare condition, with annual incidence of three to eight cases/100,000 people [1–3], which is associated with high morbidity and mortality. Type A aortic dissections are viewed as surgical emergencies, but the treatment of type B aortic dissections (TBADs), which account for about one-third of total aortic dissections [4], is more variable. Acute type B dissections that are complicated by visceral or limb ischemia, aortic rupture, refractory pain, or rapidly expanding dissection also require surgical intervention [5]. In the absence of such complications, which is the case for seventy percent of patients with acute type B dissection [6], medical therapy with antihypertensives has been the standard of care.

In 2005, the US Food and Drug Administration approved thoracic endovascular aortic repair (TEVAR) for the repair of thoracic aortic aneurysms [7], and surgeons have increasingly been using TEVAR off-label to treat TBAD. The high mortality rates associated with traditional open thoracotomy surgical repair of complicated TBAD have driven the increased adoption of the less invasive TEVAR in complicated TBAD. The possibility that TEVAR can facilitate better remodeling of the aorta than medical therapy and avert late aortic rupture has stimulated its use in treating uncomplicated TBAD patients, who traditionally would have received medical therapy alone [8, 9].

Most studies on the outcomes of TEVAR have been small, single center studies [10, 11], or meta-analyses with potential selection biases [5, 12], but Sachs et al. [13] were the first to conduct a large population study utilizing the Nationwide Inpatient Sample which compared the outcomes of treatment with TEVAR versus open surgery in TBAD patients. Our study expands on the work done by Sachs et al. [13], as we include an additional year of data (2008) and utilize a propensity score matching approach that more effectively controls for differences between treatment groups than standard multivariate analysis. Moreover, we examine the relationship between volume and outcome of TEVAR procedures.

## 2. Materials and Methods

### 2.1. Data Sources and Study Population

We used the Nationwide Inpatient Sample (NIS) database to identify patients with type B thoracic aortic dissections who underwent surgical repair between 2005 and 2008. The NIS is the largest all-payer inpatient care database and contains discharge data from over eight million hospitalizations per year, which is approximately twenty percent of all hospitalizations in the United States. 

*International Classification of Diseases, the 9th edition* (ICD-9) diagnosis codes were used to identify all patients in the NIS with thoracic aortic dissections (441.01). Patients with thoracic dissections treated surgically were separated from those receiving medical therapy alone by using procedure codes for endovascular stent graft repair (39.73) or open repair (38.34, 38.45). The ICD-9 procedure code for TEVAR was introduced in September 2005, which limited our study to the period from 2005 to 2008. Additionally, we excluded all patients with diagnosis codes for aortic aneurysms (441.1 to 441.9), to better restrict the analysis to patients with acute dissection. We isolated type B aortic dissections from type A aortic dissections using criteria developed by Sachs et al. [13], in which patients with procedure codes for cardioplegia (39.63), valve repair (35.00–35.99), or operations on vessels of the heart (36.00–36.99, 37.0, 37.2, 37.31–37.90, 37.93–37.99), who were more likely to have type A dissections, were excluded. We further stratified the patients with type B dissections based on whether they were admitted emergently or electively to the hospital.

The following patient demographics and hospital characteristics were identified: age, gender, hospital bed size, hospital teaching status, and annual volume of TEVAR performed per hospital. The following comorbidities were assessed: cardiac arrhythmias, chronic congestive heart failure (CHF), coronary artery disease, valvular heart disease, chronic pulmonary disease, chronic renal failure, coagulopathy, deficiency anemias, diabetes, hypertension, obesity, neurological disorders, paralysis, and peripheral vascular disease (Table 1).

### 2.2. Statistical Analysis

In order to control for imbalances of patient characteristics and institutional characteristics among the treatment groups that might influence treatment outcome, we used a propensity scoring method to establish matched cohorts. Separate models were created for emergent and elective admissions. A propensity score, which was assigned to each hospitalization, was based on a multivariate logistic regression model that examined the impact of thirty variables (patient demographics, comorbidities, and hospital characteristics) on the likelihood of treatment assignment. Patients with similar propensity scores in the two treatment groups were matched using a 1-to-1 scheme without replacement, using 8-to-1 digit match.

Outcomes were compared between propensity score-matched cohorts of patients undergoing TEVAR and open surgery. The primary outcomes measured were in-hospital mortality, in-hospital complications, and length of stay (LOS). Complications included acute renal failure, cardiac complications, neurological complications, paraplegia, posthemorrhagic anemia, postoperative hemorrhage, pulmonary complications, stroke, and thrombectomy/embolectomy. The ICD-9 diagnosis codes used to code for comorbidities and complications are provided in Table 1. Paired *t*-test was used for comparisons of continuous variables and the McNemar test for categorical variables in matched cohorts. All statistical analyses were performed using SAS 9.2 software (SAS Institute Inc., Cary, NC).

## 3. Results

### 3.1. Baseline Demographics and Comorbidities

During the period 2005–2008, 4752 emergently admitted patients with TBAD were treated surgically: 3427 (72.1%) underwent open repair, and 1325 (27.9%) underwent TEVAR (Table 2). During this same period of time, 1247 electively admitted patients with TBAD were treated surgically: 680 (54.5%) underwent open repair, and 567 (45.5%) underwent TEVAR (Table 3). Among those admitted emergently, TEVAR recipients were significantly more likely to have coronary artery disease, chronic pulmonary disease, diabetes, peripheral vascular disease and to be treated in hospitals with higher TEVAR volume. In contrast, open repair patients had higher rates of cardiac arrhythmia, valvular disease, and coagulopathy (Table 2). Among electively admitted patients, TEVAR recipients were older, had higher rates of diabetes, hypertension, and were more likely to receive their care at higher TEVAR volume hospitals. Open repair patients had significantly higher rates of valvular disease and coagulopathy (Table 2). 

### 3.2. Outcomes

Propensity score matching in the emergently-admitted TBAD patients produced 991 matched pairs. Demographic traits and comorbidities were balanced between these matched cohorts (Table 2). The *P* value for the Hosmer-Lemeshow goodness-of-fit test of the propensity score model was 0.605, and the *c* statistic was 0.828. In-hospital mortality among emergently admitted patients was significantly higher among open repair recipients than in TEVAR recipients (17.5% versus 10.8%, *P* =  .045). Open repair was associated with higher rates of acute renal failure (27.3% versus 16.8%, *P* =  .008), cardiac complications (16.6% versus 8.9%,* P* =  .029), and posthemorrhagic anemia (22.6% versus 12.1%, *P* =  .006) (Table 4). 

Propensity score matching in electively admitted TBAD patients produced 282 matched pairs. Demographic characteristics and comorbid conditions were similar between these two matched cohorts (Table 3). The *P* value for the Hosmer-Lemeshow goodness-of-fit was 0.137, and the *c* statistic was 0.858. In-hospital mortality was not significantly different among recipients of TEVAR and open surgery (5.6% versus 3.3%, *P* =  .464). Open repair recipients did have higher rates of in-hospital complications such as acute renal failure (13.0% versus 1.7%, *P* =  .021), cardiac complications (15.8% versus 3.6%, *P* =  .021), posthemorrhagic anemia (17.3% versus 3.5%,* P* =  .017), postoperative hemorrhage (19.1% versus 5.2%, *P* =  .009), and pulmonary complications (35.0% versus 16.2%,* P* =  .034) (Table 5). 

### 3.3. TEVAR Volume-Mortality Relationship

**Figure 1:**
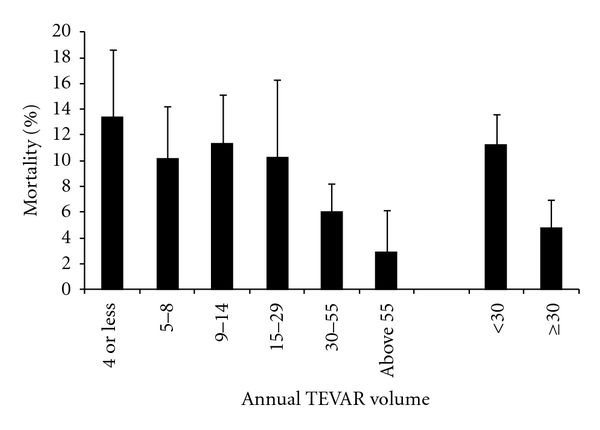
In-hospital mortality rates for emergently admitted TBAD patients undergoing TEVAR based on the annual hospital volume of TEVAR procedures performed. The following are the number of emergently admitted TBAD patients treated with endovascular repair in each TEVAR volume group: 1–4 (*n* = 225), 5–8 (*n* = 237), 9–14 (*n* = 217), 15–29 (*n* = 239), 30–55 (*n* = 241), greater than 55 (*n* = 167), less than 30 (*n* = 917), 30 or more (*n* = 408).

To determine whether outcomes of TEVAR treatment for TBAD are related to overall hospital experience with this procedure (i.e., for aneurysms and dissection), we grouped hospitals by overall TEVAR volume and compared in-hospital TBAD mortality rates. We limited the analysis to TBAD patients admitted emergently to help control for patient differences (Figure 1). The cut-off volumes for the six TEVAR volume groups were established so that each group would have a similar number of emergently admitted TBAD patients treated with TEVAR. The lowest volume group (1–4 TEVAR procedures/year) has the highest mortality rate (13.4%). The highest volume group (over 55 TEVAR procedures/year) has the lowest mortality rate (2.9%). The TBAD mortality rate, which was relatively uniform at lower volumes, declined steadily as hospital TEVAR volumes reached 30 per year. In fact the TBAD in-hospital mortality rate for hospitals performing less than 30 procedures per year was more than twice the rate seen in hospitals that performed more than 30 per year (<30 = 11.3%, ≥30 = 4.8%; *P *=  .047).

## 4. Discussion

We looked at TEVAR and open repair use for type B aortic dissection over a four-year period and compared outcomes. To control for selection bias we used a propensity score model to create matched cohorts of patients for the comparisons. Among patients treated emergently, in-hospital mortality was significantly lower with TEVAR than open surgery (10.8% versus 17.5%, *P* =  .045). This finding is consistent with other reports in the literature. In patients with complicated TBAD, Zeeshan et al. [14] demonstrated significantly lower 30-day mortality (4% versus 40%; *P *=  .006) among TEVAR recipients (*n* = 45) compared to open repair recipients (*n* = 20). Also, in patients with complicated TBAD, International Registry of Acute Aortic Dissection (IRAD) investigators demonstrated significantly lower in-hospital mortality among TEVAR recipients (10.6% versus 33.9%, *P* =  .002) as well as lower rates of stroke and acute renal failure (20.8% versus 40.0%, *P *=  .04) [6]. In a recent meta-analysis of TEVAR use in complicated acute type B dissections, Luebke and Brunkwall [15] reported an in-hospital mortality rate of 11.5%.

In contrast to what we observed with emergently admitted patients, TEVAR did not appear to have an impact on the mortality of nonemergent TBAD patients (3.3% versus 5.6%, *P *=  .464). However, the more relevant question for patients with uncomplicated type B aortic dissection is how the outcomes of TEVAR compare to medical therapy, the standard of care for this disease. Unfortunately, our study could not address this question as large datasets like the NIS do not contain anatomical information about the disease, which would be needed to distinguish type A and type B dissections. Our ability to make this distinction in surgical patients relied on the coding of associated aortic arch and aortic valve procedures, commonly required to repair type A dissections. It is clear that comparing the outcomes of medical therapy to TEVAR for uncomplicated type B dissections will require primary data collection, preferably in the form of a randomized controlled trial.

In looking at the relationship between TEVAR volume and outcome of treatment in emergently treated TBAD patients, we observed over a fourfold difference in mortality (13.4% versus 2.9%) between the lowest volume group (1–4 TEVAR procedures/year) and the highest volume group (over 55 TEVAR procedures/year). Hospitals that perform less than thirty TEVAR procedures annually had more than twofold higher in-hospital mortality for emergent TBAD patients than hospitals performing more than 30 TEVAR procedures a year (11.3% versus 4.8%, *P *=  .047). Although this analysis did not adjust for differences between the volume groups, which would have required a larger sample size, our restricting the analysis to the subgroup who was hospitalized emergently does help to minimize the differences between the volume groups. Our finding is consistent with others. In a meta-analysis of 609 patients undergoing TEVAR for aortic dissection (96% TBAD), Eggebrecht et al. [5] concluded that surgeons' experience with TEVAR influenced outcomes because hospitals that performed more than twenty TEVAR procedures had significantly lower 30-day mortality than hospitals with less than twenty procedures (3.2% versus 8.5%; *P *<  .001).

One limitation of our study is that it is observational and carries all the potential biases inherent to such studies. For instance, the subgroups of patients who underwent TEVAR and open repair differed substantially (Tables 2 and 3). We adjusted our comparisons for such differences by comparing propensity score-matched cohorts; however, it is possible that there is still some bias in the analyzed cohorts due to confounders that were not recognized. Furthermore, the comorbidity data, which was the basis for the comparisons between treatment groups, was originally collected for administrative purposes and subject to coding errors. Another important limitation to our study is that it does not address long-term outcomes. Complications unique to treatment with TEVAR, such as endoleak, graft migration, and retrograde type A dissection, can contribute to mortality and complications after a patient is discharged from the hospital [16, 17]. Longitudinal analysis in the form of randomized controlled clinical trials is needed in order to determine the long-term outcomes of TEVAR used for type B dissections and to ascertain whether favorable in-hospital outcomes for TEVAR in emergently admitted TBAD patients persist over time. The recent INSTEAD trial was the first prospective, randomized, and controlled comparison of TEVAR and medical treatment in uncomplicated TBAD, and it showed that there was no difference in one-year mortality between TEVAR and medical therapy alone, despite significantly greater aortic remodeling in TEVAR [18]. However, INSTEAD was not appropriately powered to find a difference with the high survival rates they observed in the medical therapy group. Moreover, INSTEAD did not restrict enrollment to current definitions of acute uncomplicated TBAD, which many believe is the subgroup with greatest chance of showing a benefit to TEVAR [8]. Many of these limitations are being addressed by the ongoing ADSORB clinical trial [19].

## 5. Conclusions

Our study demonstrates that endovascular repair is becoming an important treatment modality for type B dissection. Approximately 31.5% of the surgical repairs of TBAD in the United States are performed using this modality. Our analysis of the NIS dataset, using propensity score matching, demonstrated that TEVAR outperforms open surgery among emergently admitted patients with type B aortic dissection, both in terms of in-hospital mortality and morbidity. This was not the case for electively admitted patients, where TEVAR appeared to impart a short-term morbidity benefit only. We observed a significant volume outcome relationship between overall TEVAR use and in-hospital mortality rates, which, if confirmed through additional studies, should guide institutional targets for optimal outcomes of therapy.

## Figures and Tables

**Table:** (a)

Comorbidity	ICD-9 code
Cardiac arrhythmia	426.0, 426.10, 426.11, 426.12, 426.13, 426.7, 426.9, 427.0, 427.1, 427.2, 427.3, 427.9, V45.0, V53.3
Chronic CHF	398.91, 402.01, 402.11, 402.91, 404.01, 404.03, 404.11, 404.91, 404.13, 404.93, 425.4, 425.5, 425.7, 425.8, 425.9, 428.0, 428.1, 428.20, 428.22, 428.30, 428.32, 428.40, 428.42, 428.9
Coronary disease	412, 413, 414, 429.2
Valvular disease	093.2, 394, 395, 396, 397, 424, 746.3, 746.4, 746.5, 746.6, V42.2, V43.3
Cardiac comorbidities	Any of the codes for cardiac arrhythmia, chronic CHF, coronary disease, or valvular disease
Chronic pulmonary disease	416, 417.9, 490, 491, 492, 493, 494, 495.0, 495.1, 495.2, 495.3, 495.4, 495.5, 495.6, 495.8, 495.9, 496, 500, 501, 502, 503, 504, 505, 506.0, 506.2, 506.4, 506.9, 508.1, 508.8, 508.9
Chronic renal failure	403.01, 403.11, 403.91, 404.02, 404.03, 404.12, 404.13,404.92, 404.93, 585, 586, V42.0, V45.1, V56.0–V56.2, V56.8
Coagulopathy	2860–2869, 287.1, 287.3–287.5, 289.81–289.82
Deficiency anemias	280.1–281.9, 285.21–285.29, 285.9
Diabetes	250
Hypertension	401.0, 401.1, 401.9, 402.00, 402.10, 402.90, 403.00, 403.10, 403.90, 404.00, 404.10, 404.90, 405.01, 405.09, 405.11, 405.19, 405.91, 405.99, 462.24, 642.00–642.04, 642.10, 642.70–642.94
Obesity	278.0, 278.00, 278.01, V85.30, V85.31, V85.32, V85.33, V85.34, V85.35, V85.36, V85.37, V85.38, V85.39, V85.4, V85.54
Other neurological disorders	330.0–331.9, 332.0, 333.4, 333.5, 334.0–335.9, 333.71, 333.72, 333.79, 333.85, 333.94, 338.0, 340, 341.1–341.9, 345.00–345.11, 345.2–345.3, 345.40–345.91, 34700, 34701, 34710, 34711, 348.1, 348.3–348.39, 780.3, 780.39, 784.3
Paralysis	342.0–342.12, 342.9–344.9, 438.20–438.53
Peripheral vascular disease	440.0–440.9, 441.00–441.9, 442.0–442.9, 443.1–443.9, 444.21, 444.22, 449, 447.1, 557.1, 557.9, V43.4

**Table:** (b)

Complication	ICD-9 code
Acute renal failure	584
Cardiac complications	410.00, 410.01, 410.10, 410.11, 410.20, 410.21, 410.30, 410.31, 410.40, 410.41, 410.50, 410.51, 410.60, 410.61, 410.70, 410.71, 410.80, 410.81, 410.90, 410.91, 411.0, 411.1, 411.81, 411.89, 427.5, 428.21, 428.3, 428.31, 428.32, 428.41, 428.43, 997.1
Neurological complications	780.01 344.1 997.02 997.0 435 436, 437.1
Paraplegia	344.1
Posthemorrhagic anemia	285.1
Postoperative hemorrhage	998.1
Pulmonary complications	311, 312.9, 415.0, 415.11, 415.12, 415.19, 481, 482.0, 482.1, 482.2, 482.30, 482.31, 482.32, 482.39, 482.81, 482.82, 482.83, 482.84, 482.89, 482.9, 485, 486, 518.0, 518.4, 518.82, 518.84, 528.81, 997.3
Stroke	997.02
Thrombectomy or embolectomy	38.03, 38.04, 38.06, 38.08

**Table 2:** Baseline characteristics of emergently admitted patients undergoing open surgical repair or TEVAR for type B dissections from 2005 to 2008.

Variable	Unmatched cohort				Matched cohort		
	Open repair (*n* = 3427)	TEVAR (*n *= 1325)	*P* value		Open repair (*n* = 991)	TEVAR (*n* = 991)	*P* value

	Mean or % of patients				Mean or % of patients		

Female sex	37.2	38.9	0.642		35.5	40.4	0.309

Age							
Mean age (years)	59.9	61.2	0.153		61.9	61.0	0.429
<60 yr	47.3	39.8	0.027		37.0	38.1	0.826
60–64 yr	10.5	12.9	0.302		12.2	12.8	0.868
65–69 yr	11.5	10.6	0.701		14.1	12.2	0.576
70–74 yr	9.9	11.9	0.393		13.0	13.5	0.890
75–79 yr	11.0	13.8	0.230		13.8	12.8	0.782
≥80 yr	9.8	11.1	0.511		9.9	10.7	0.751

Hospital bed size							
Small	3.4	16.5	<0.001		5.5	7.2	0.550
Medium	14.9	12.0	0.446		13.6	15.4	0.683
Large	81.7	71.6	0.144		80.9	77.4	0.468

Teaching hospital	73.6	74.3	0.933		82.2	79.3	0.518

Mean annual TEVAR volume (*n*)	8.2	27.3	<0.001		15.1	15.4	0.894

Comorbid conditions							
Cardiac comorbidities	54.9	39.9	<0.001		44.9	42.1	0.558
Cardiac arrhythmia	34.1	14.8	<0.001		15.3	16.8	0.648
Chronic CHF*	11.8	9.2	0.186		9.3	9.5	0.940
Coronary disease	10.1	17.5	0.004		19.4	17.0	0.556
Valvular disease	20.3	10.7	0.003		12.7	12.8	0.976
Chronic pulmonary disease	15.0	23.9	<0.001		25.3	24.0	0.755
Chronic renal failure	10.0	10.9	0.679		7.8	10.1	0.346
Coagulopathy	19.9	8.1	<0.001		9.0	10.3	0.666
Deficiency anemias	15.4	14.3	0.674		16.8	15.5	0.693
Diabetes	7.7	12.3	0.012		10.8	11.9	0.691
Hypertension	65.8	66.0	0.950		65.8	66.3	0.922
Obesity	7.1	4.6	0.142		3.9	4.4	0.782
Other neurological disorders	5.1	3.1	0.198		2.5	2.8	0.876
Paralysis	5.4	5.1	0.838		5.3	6.2	0.687
Peripheral vascular disease	14.9	32.6	<0.001		25.6	25.7	0.980

*CHF: congestive heart failure.

**Table 3:** Baseline characteristics of electively admitted patients undergoing open surgical repair or TEVAR for type B dissections from 2005 to 2008.

Variable	Unmatched cohort				Matched cohort		
	Open repair (*n* = 680)	TEVAR (*n* = 567)	*P* value		Open repair (*n* = 282)	TEVAR (*n* = 282)	*P* value

	Mean or % of patients				Mean or % of patients		

Female sex	29.5	25.9	0.520		32.0	26.6	0.531

Age							
Mean age (years)	58.3	63.0	0.008		60.7	62.0	0.485
<60 yr	50.4	37.2	0.040		46.1	44.3	0.842
60–64 yr	12.2	14.0	0.637		11.8	11.9	0.992
65–69 yr	11.9	14.7	0.548		10.8	12.4	0.822
70–74 yr	11.0	19.1	0.080		15.9	17.5	0.822
75–79 yr	10.2	11.0	0.852		8.6	8.7	0.978
≥80 yr	4.3	4.1	0.943		6.9	5.3	0.758

Hospital bed size							
Small	4.4	17.3	0.022		7.9	9.3	0.739
Medium	18.5	16.1	0.743		19.5	19.9	0.970
Large	77.1	66.6	0.278		72.6	70.8	0.863

Teaching hospital	79.6	75.4	0.632		79.1	85.7	0.483

Mean annual TEVAR volume (*n*)	9.3	28.5	<0.001		14.1	14.7	0.656

Comorbid conditions							
Cardiac comorbidities	53.1	39.6	0.048		42.2	37.5	0.635
Cardiac arrhythmia	18.8	17.0	0.700		23.1	16.6	0.323
Chronic CHF*	11.1	10.2	0.828		12.2	11.9	0.973
Coronary disease	16.2	23.1	0.233		15.9	16.7	0.916
Valvular disease	27.9	7.3	<0.001		8.3	11.8	0.504
Chronic pulmonary disease	18.2	23.6	0.309		19.5	24.4	0.496
Chronic renal failure	12.7	11.4	0.754		10.5	15.5	0.445
Coagulopathy	15.7	3.7	0.004		3.5	5.1	0.681
Deficiency anemias	10.2	8.4	0.632		10.5	12.0	0.801
Diabetes	9.8	19.6	0.030		10.7	14.0	0.554
Hypertension	65.4	85.0	<0.001		72.0	76.0	0.600
Obesity	8.3	13.9	0.129		11.0	8.6	0.690
Other neurological disorders	4.4	3.8	0.793		1.9	3.9	0.566
Paralysis	3.0	0.9	0.269		1.7	1.8	0.967
Peripheral vascular disease	19.7	27.6	0.131		21.3	27.7	0.409

*CHF: congestive heart failure.

**Table 4:** In-hospital outcomes after open repair or TEVAR in a matched cohort of emergently admitted patients.

	Open repair (*n* = 991)	TEVAR (*n* = 991)	*P* value

	mean or % of patients		

Mortality	17.5	10.8	0.045
Acute renal failure	27.3	16.8	0.008
Cardiac complications	16.6	8.9	0.029
Neurological complications	11.0	9.3	0.561
Paraplegia	2.5	3.2	0.705
Posthemorrhagic anemia	22.6	12.1	0.006
Postoperative hemorrhage	13.5	9.2	0.209
Pulmonary complications	34.4	24.9	0.052
Stroke	6.2	3.7	0.239
Thrombectomy or embolectomy	1.9	5.0	0.133
Mean length of stay (days)	15.0	13.9	0.832

**Table 5:** In-hospital outcomes after open repair or TEVAR in a matched cohort of electively admitted patients.

	Open repair (*n* = 282)	TEVAR (*n* = 282)	*P* value
	mean or % of patients		

Mortality	5.6	3.3	0.464
Acute renal failure	13.0	1.7	0.021
Cardiac complications	15.8	3.6	0.021
Neurological complications	1.9	5.4	0.348
Paraplegia	0.0	0.0	
Posthemorrhagic anemia	17.3	3.5	0.017
Postoperative hemorrhage	19.1	5.2	0.009
Pulmonary complications	35.0	16.2	0.034
Stroke	1.9	3.6	0.600
Thrombectomy or embolectomy	1.8	7.3	0.171
Mean length of stay (days)	11.3	7.7	0.205
